# High porosity 3D printed titanium mesh allows better bone regeneration

**DOI:** 10.1186/s12903-023-02717-5

**Published:** 2023-01-06

**Authors:** Rui Ma, Qian Liu, Libo Zhou, Lingxiao Wang

**Affiliations:** 1 Department of Dental Implant Centre, Beijing Stomatological Hospital, Capital Medical University, Capital Medical University School of Stomatology, No. 4 Tian Tan Xi Li, Dongcheng District, Beijing, 100050 China; 2 Beijing Citident Hospital of Stomatology, Beijing, 100032 China; 3 Digital Mesh Beijing Technology Co., Ltd, Beijing, 101312 China; 4 Heilongjiang Key Laboratory of Oral Biomedical Materials and Clinical Application, Experimental Center for Stomatology Engineering, Jiamusi University Affiliated Stomatological Hospital, Jiamusi, 154000 Jiamusi China

**Keywords:** 3D printed, Titanium mesh, Bone defects, Porosity, Oral surgery

## Abstract

**Background:**

Most patients with insufficient bone mass suffer from severe horizontal or vertical bone defects in oral implant surgery. The purpose of this study was to compare the bone regeneration effects of titanium meshes with different porosity in the treatment of bone defects.

**Methods:**

Nine beagle dogs were equally divided into three groups based on execution time. Three months after the extraction of the first to fourth premolars of the mandible, three bone defects were randomly made in the mandible. Bone particles and three kinds of three-dimensional (3D) printed titanium nets with different porosities (low porosity group (LP), 55%; medium porosity group (MP), 62%; and high porosity group (HP), 68%) were replanted in situ. The beagles were killed 4, 8, and 12 weeks after surgery. Formalin-fixed specimens were embedded in acrylic resin. The specimens were stained with micro-CT, basic fuchsin staining, and toluidine blue staining.

**Results:**

Micro-CT analysis showed that the trabecular thickness, trabecular number, and bone volume fraction of the HP group were higher than those of the other two groups. Moreover, the trabecular separation of the HP group decreased slightly and was lower than that of the MP and LP groups. Histological staining analysis showed that the trabecular number in the HP group was higher than in the other two groups at 8 and 12 weeks, and the bone volume fraction of the HP was higher than that in the other two groups at 12 weeks. Moreover, the trabecular thickness of the MP was higher than that of the LP group at 12 weeks and the trabecular separation was lower in the HP group at 4 and 8 weeks. The differences were statistically significant (*p* < 0.05).

**Conclusion:**

A 3D printed titanium mesh with HP in a certain range may have more advantages than a titanium mesh with LP in repairing large bone defects.

## Background

In oral implant surgery, most patients with insufficient bone mass suffer from severe horizontal or vertical bone defects. The majority of those patients have severe alveolar bone atrophy or defects caused by congenital tooth loss or trauma [1, 2]. One of the key factors for the success of the implant surgery is to restore the appropriate width and height of the bone and treat the large-area bone defect [3]. In the late 1960s, the titanium mesh, which is different from the traditional barrier membrane (such as the absorbable collagen membrane and the nonabsorbable expanded polytetrafluorethylene membrane), was first used in the treatment of alveolar ridge defects and achieved good clinical efficacy [3]. Moreover, the titanium mesh structure can provide good mechanical support and sufficient space for bone regeneration, allowing for the growth and development of new bone. It also has good clinical efficacy for bone reconstruction in patients with large-area or multi tooth jaw defects [4–6]. After placing the titanium mesh at the position of bone defect, it is important to assess the strength of the titanium mesh and the blood supply. The pressure generated by the labiobuccinator muscle movement or chewing of the patient will act on the titanium mesh. Accordingly, the titanium mesh must have a certain compressive capacity that prevents its collapse or shift to provide sufficient space and mechanical support for the growth of new bone. A good blood supply is conducive for the metabolism of bone marrow mesenchymal stem cells, growth factors, and other substances beneficial to osteogenesis [7]. The type of material, thickness, pore size, and porosity are all important factors in the design process of the titanium mesh. These parameters mainly affect the strength of the titanium mesh, the maintenance of blood supply to the bone, bone regeneration, and soft tissue healing. Previous studies have reported a large number of animal experiments and clinical observations on the material, thickness, and pore size of the titanium mesh, but only few studies have examined the porosity of the titanium mesh. In particular, there is a scarcity of studies reporting large-scale animal experiments that accurately observe the effect of titanium mesh porosity [8, 9]. With the recent wide application of three-dimensional (3D) printing technology in the field of oral implants, a personalized titanium mesh that fits the anatomical shape of the alveolar ridge can be manufactured. However, such a mesh is more suitable for complex jaw loss-related cases compared with the traditional titanium mesh. The size and volume of the jaw defect can be accurately calculated and analyzed through accurate computer-aided design/computer-aided manufacturing (CAD/CAM) measurements and other technologies. Moreover, a mesh structure that accurately fits the anatomy of the jaw can be designed [10]. A personalized printed titanium mesh has a good shape, a simple operation procedure, and reduces stress on the jaw bones [11–13]. Therefore, it is important to explore and clarify the appropriate range of mesh porosity during the design and manufacture processes of the titanium mesh. This study thus aimed to explore the effect of porosity of 3D printed personalized titanium meshes on the repair of alveolar bone defects in large animals using beagle dogs as an animal model to provide a reference for the future design of titanium meshes.

## Methods

This study was carried out with the approval of the Capital Medical University Animal Experiments and Experimental Animals Management Committee (Beijing, China. Approval No. AEEI-2017-104) and complied with the ARRIVE (Animal Research: Reporting of In Vivo Experiments) guidelines for preclinical animal studies. All methods were performed in accordance with the relevant guidelines and regulations.

### Experimental animals

Nine beagle dogs (1–2 years old, 15 kg) were obtained from the Institute of Animal Science, Chinese Agriculture University, China. They were housed in individual cages in a controlled environment (20–25 °C and relative humidity 40–60%). The animals were fed a soft dog-food diet (Science Diet, Hill’s Pet Co., Topeka, KS, USA) and had free access to water.

### Surgical procedure

Veterinary assistance was used throughout the procedures, and all efforts were taken to minimize pain. In the first stage of surgery, all animals were preanesthetized with atropine sulfate (0.05 mg/kg intramuscular (IM) injection; Guangdong South China Pharmaceutical Co., Ltd., Guang Dong, China) and tiletamine/zolazepam (5 mg/kg IV; Zoletil 50; Virbac, Carros, France). Local anesthesia induction was achieved by infiltrating 1 mL of articaine (Produits Dentaires Pierre Rolland, France) containing epinephrine (1:100,000) into the mucosa at the surgical sites. Anesthesia was sustained with sevoflurane (Sevorane; Capital Medical University, Beijing, China). The buccal, lingual, and lateral walls of the alveolar sockets were preserved. The mandibular first to fourth premolars were carefully extracted without damaging the extraction sockets. Multimodal analgesia was used during the perioperative period. After tooth extraction, metacam 0.1 mg/kg (PO; Boehringer Ingemheim Co., Ridgefield, CT, USA), ketorolac 1 mg/kg (Toradol 30 mg; Shanghai Roche Pharmaceuticals Co., Ltd., Shang Hai, China), tramadol 1.7 mg/kg (Ado-lonta injectable, Grünenthal, Huayou medical group, Beijing, China), and buprenorphine 0.01 mg/kg (Buprex; Reckitt Benckiser Pharmaceuticals Limited, Berkshire, UK) were used to reduce pain. To prevent postoperative infection, amoxicillin (20 mg/kg PO; The United Laboratories Co., Ltd., Hong Kong, China) was administered for 6 days. Each dog was fed a liquid diet for 2 weeks, followed by a soft diet. The extraction sites were allowed to heal for 3 months.

**Fig. 1:**
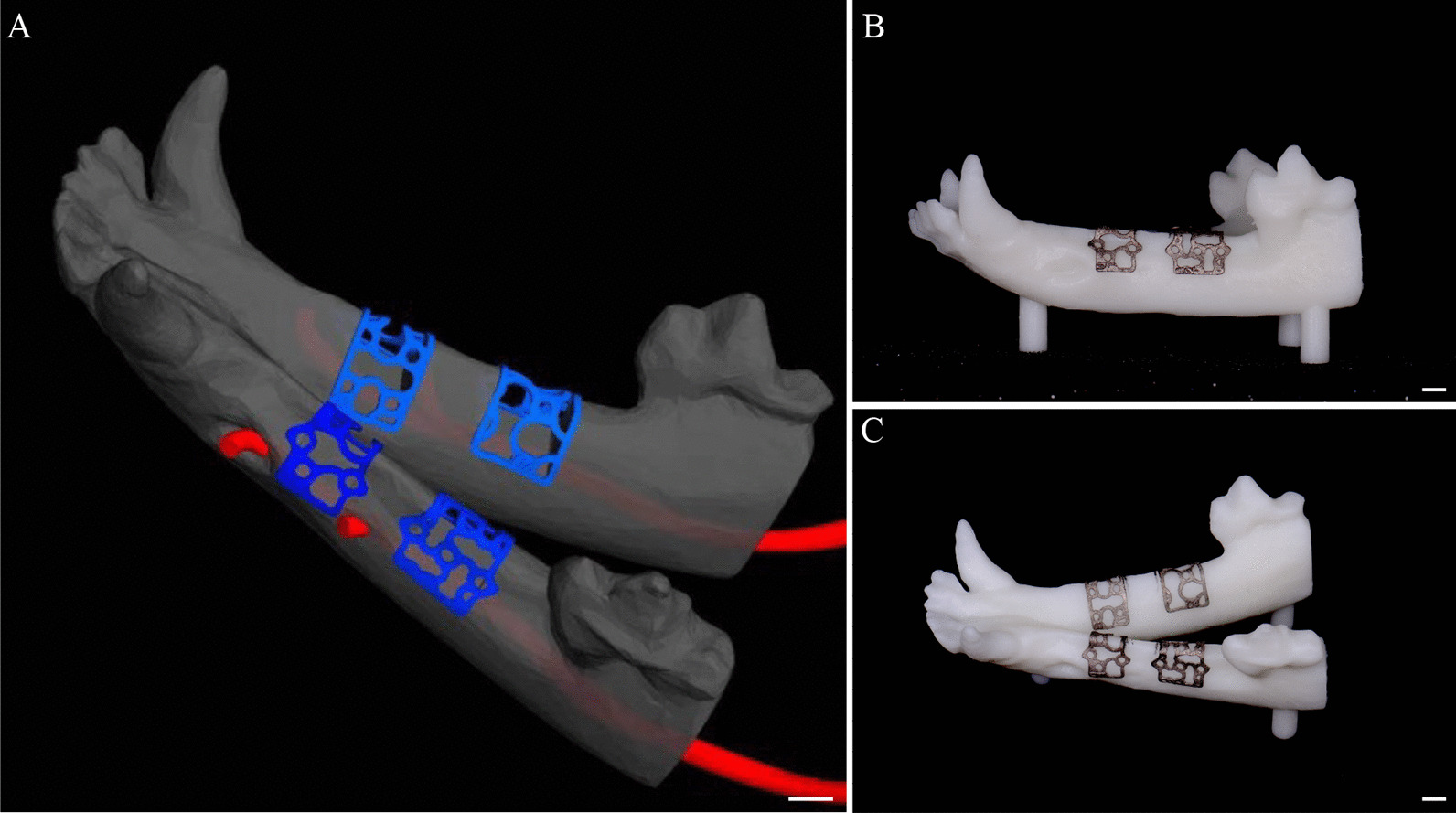
The 3D reconstruction model of the mandible of a beagle dog and the 3D printed titanium mesh. **A** Design of the personalized 3D printed titanium mesh in 3-matic research 11.0 software; **B**, **C** the 3D printed canine mandible model and personalized titanium mesh. Scalebar = 1.5 mm

**Fig. 2:**
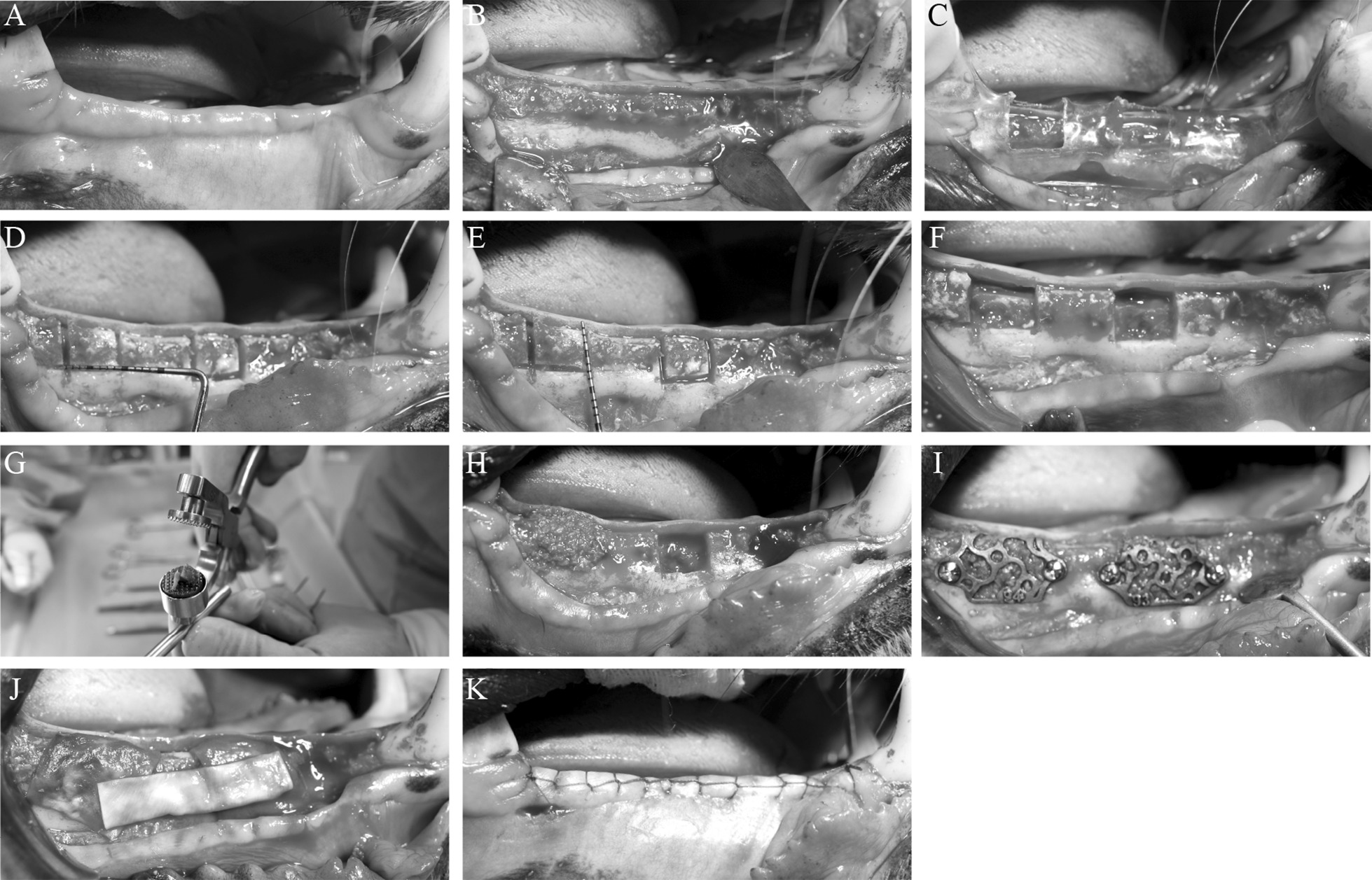
Bone defect establishment process and titanium mesh implantation model. **A** Three months after tooth extraction, the mucosa of the beagle dogs healed well. The next steps were as follows: **B** open the flap; **C** place the simple osteotomy guide plate; **D**, **E** measure the range of the bone defect area; **F** induce a bone defect using an ultrasonic bone knife; **G** crush the removed bone pieces into bone particles; **H** perform in situ replantation of the bone granules; **I** place the titanium mesh; **J** cover the biofilm; and **K** close and stitch the flap

After the tooth extraction socket of the beagle dogs healed, the maxillofacial region of the beagle dogs was scanned with Cone beam Computer Tomography (CBCT). The CBCT image data were obtained and saved in DICOM format. The digital files were sent to the Ti-mesh manufacturer (Digital Mesh Technology Co., Ltd, Beijing China) to create a 3D copy of the edentulous site and the adjacent teeth (Fig. 1A). The thickness of the titanium mesh design was 0.4 mm, the diameter of the circular aperture was 1.4 mm, and the porosity was set at three different percentages: 55%, 62%, and 68% for low porosity (LP), medium porosity (MP), and high porosity (HP), respectively. The geometric basic structure of the titanium mesh is a spindle-shaped cell, with an average pore diameter of 1.4 ± 0.05 mm. The side lengths of the cells of the different porosity groups LP, MP, and HP are 8 ± 0.1 mm, 10 ± 0.1 mm, and 12 ± 0.1 mm, respectively. This model was used for the individual CAD of the digital Ti-mesh. After approval by the surgeon, class IV titanium mesh was produced by means of selective laser sintering (Yxoss CBR^®^) (CAM). After the selective laser sintering process, the component underwent a blasting surface treatment process (Fig. 1B, C). Finally, ultrasonic washing, sterilization, and disinfection were carried out. After completion, sterile packaging was used to seal it. In the second stage, the mandible model of each beagle dog was printed according to their respective CBCT. The mandible model was then measured and marked according to the distance between the bone defect design site and the canine and molar teeth and the distance between the two bone defect sites. A simple pressed film guide plate was made to unify the position, size, and shape of the bone defect during the operation. Anesthesia and perioperative management procedures were the same as those carried out in the first stage. A horizontal incision was made along the alveolar crest, ranging from the mesial region of the mandibular molar to the distal region of the canine. The buccal and lingual mucoperiosteal membranes were peeled off and the flap was turned over to expose the bone surface. Two bone defects were created on the buccal side in each quadrant with an ultrasonic osteotome according to the osteotomy guide plate. The depth, mesial distal direction, and buccolingual direction were 6 mm apart with 10 mm intervals. The autologous bone blocks taken out were ground into granules and implanted into the bone defect area. Three bone defect sites were made for each beagle dog and were randomly allocated according to the porosity groups (55%, 62%, and 68% for the LP group, MP group, and HP group, respectively). Thereafter, the sites were implanted with a personalized titanium mesh and Bio-Gide barrier membrane (Geistlich, Switzerland). The tension was reduced, and the absorbable thread (vycril 5/0) was pulled for creating tight sutures. At weeks 4, 8, and 12, three dogs were euthanized by administering concentrated sodium pentobarbital IV (Euthasol, Delmarva Laboratories, Inc., Midlothian, VA, USA). The block sections were immediately collected from the alveolar bone (Fig. 2A–K).

### Micro-computed tomography (micro-CT) evaluation

The specimens were scanned on a micro-CT system (Inveon, Siemens, Germany; 80 kV, 500 µA, 1500 ms exposure time) before mechanical and histological assessment. The trabecular thickness (Tb. Th), trabecular number (Tb. N), trabecular separation (Tb. Sp), bone surface/bone volume ratio (BS/BV), and bone volume/total volume ratio (BV/TV) were determined.

### Histochemical examination

The specimens were fixed with 10% formalin, decalcified with 10% ethylenedi-aminetetraacetic acid (pH 7.0), and dehydrated through a graded series of ethanol solutions and 100% acetone. Thereafter, the specimens were embedded in methyl methacrylate. The tissue slides (25 μm) were prepared in the buc-colingual direction parallel to the axis of the implants by an EXAKT 400CS grinding machine (Leica, Wetzlar, Germany). The slides were then stained with the toluidine blue–basic fuchsin stain. Images were captured and analyzed by a light microscope (Olympus BH2 with S Plan FL2 lens, Tokyo, Japan) and a computer-digitized image analysis system (Leica Imaging System, Cambridge, England). The Tb. Th, Tb. N, Tb. Sp, BS/BV, and BV/TV were determined.

### Statistical analysis

All statistical calculations were performed with SPSS 16 statistical software (SPSS, Inc., Cary, NC, USA). Statistical significance was determined by independent-samples Student’s *t*-test or analysis of variance (ANOVA). The *p*-values less than 0.05 were considered statistically significant; *p* < 0.05 (*), *p* < 0.001 (**), *p* < 0.0001 (****).

## Results

### Successful titanium mesh construction and animal model development

We successfully designed the personalized 3D printed titanium mesh in 3-matic research 11.0 software and printed the 3D reconstruction model and titanium mesh of the beagles’ mandible (Fig. 1A, B and C). Three months after tooth extraction, the mucosa of the 9 beagles healed well. We successfully established the jaw bone defect model of 9 beagle dogs and implanted the titanium mesh (Fig. 2A–K). The healing process of all animals was smooth, and no postoperative complications were observed in the 12 week observation period. After 3–6 days of rest, the animals could move freely without any obvious pain or restriction.

### Micro-CT revealed better bone reconstruction in the HP group than in the MP and LP groups

**Fig. 3:**
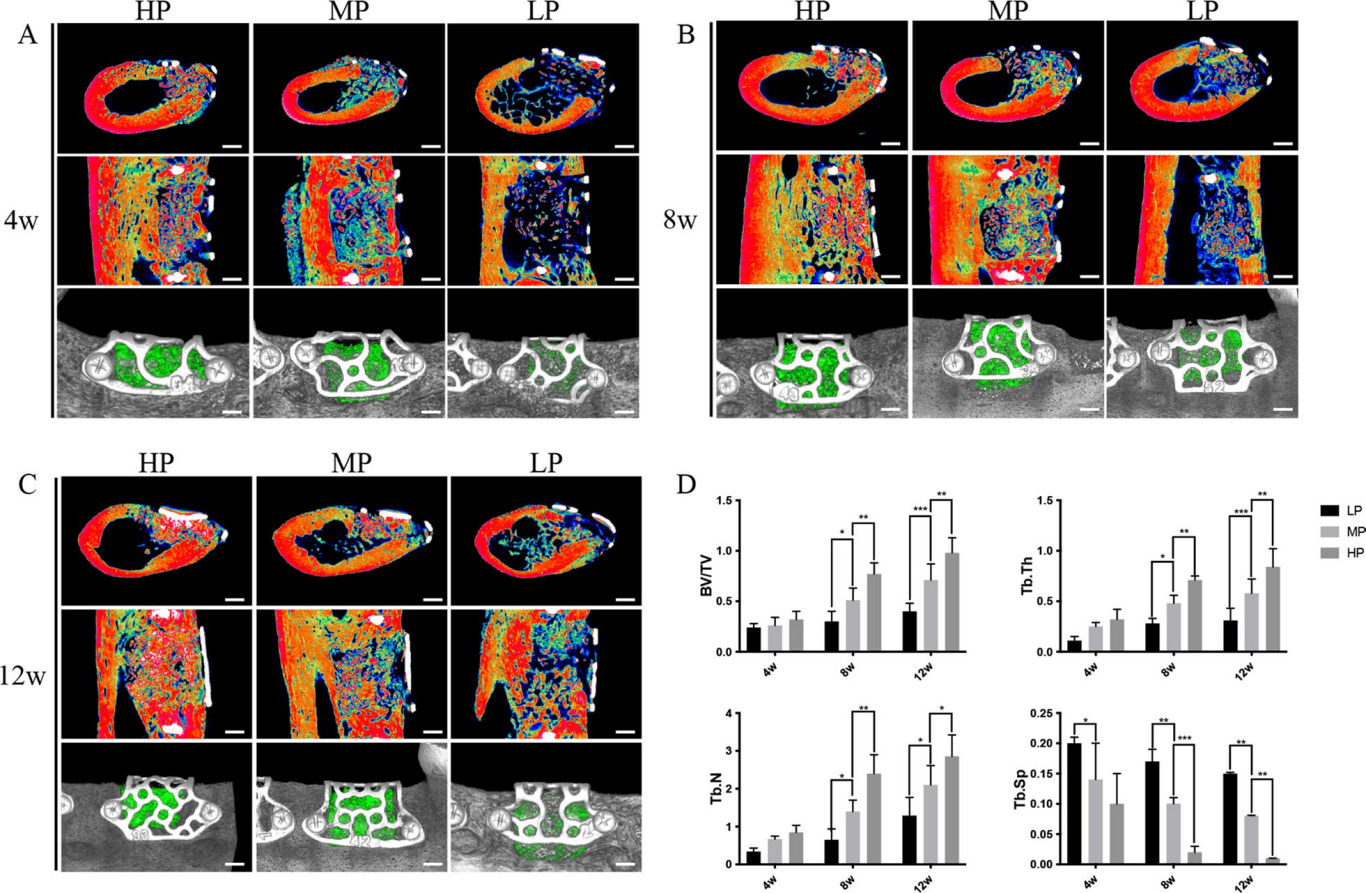
Micro-CT image results of the repair effect of different titanium meshes on bone defects. **A** After 4 weeks, the bone defect area can be observed in the coronal and sagittal positions. In the HP group, mixed masses of old and new bone with low density are observed. In the MP group, old bone and fibrous tissue are noted. In the LP group, old bone particles left in the bone defect area are observed. After three-dimensional reconstruction, the volume of the new bone was larger in the HP group followed by MP and LP groups; **B** After 8 weeks, the bone defect area in each group was repaired to a certain extent compared with that at 4 weeks. From the perspective of coronal, sagittal, and three-dimensional reconstruction, the new bone density and volume had the same pattern as that after 4 weeks; **C** After 12 weeks, the bone defect area in each group was further repaired compared with that at 8 weeks, especially in the HP group. The old and new bones were combined, as observed in the coronal and sagittal positions, filling the whole bone defect area; **D** Parameter analysis showed that Tb. Th, Tb. N, and BV/TV in each group increased with the increase of bone healing time, while Tb. SP decreased with the increase of bone healing time; thus, the new bone mineral density showed an upward trend. At each time point, Tb. TH, Tb. N, and BV/TV in the HP group were higher than those in the other two groups, and Tb. SP decreased slightly with time and was lower than that in the other two groups. In the MP group, BV/TV, Tb. TH, and Tb. N were higher than those in the LP group, while Tb. SP was lower than that in the LP group, and a statistical difference between them was observed. Scalebar = 1.5 mm

Micro-CT evaluation showed that the titanium mesh with HP formed more new bones. The sagittal and coronal schematic diagrams of the region of interest and the volume of new bones after 3D reconstruction were evaluated. In the longitudinal comparison of the time axis, the bone defects of the titanium mesh in the 4, 8, and 12 week groups had different degrees of new bone formation. In the transverse comparison, the effect of new bone formation at each time node was higher with increasing porosity (HP group > MP group > LP group; Fig. 3A, B and C). In the longitudinal direction of the time axis of the parameter analysis, Tb. Th, Tb. N, and BV/TV in the bone defect area increased with an increase in the bone healing time. On the other hand, Tb. SP decreased with an increase in the bone healing time, indicating that the density of the new bone was increasing. In the horizontal comparison, the Tb. Th, Tb. N, and BV/TV of the titanium mesh in the HP group were higher than those in the other two groups. The Tb. SP decreased slightly and was lower than in the other two groups. The BV/TV, Tb. Th, and Tb. N in the MP group were statistically higher than those in the LP group, while the Tb. SP of the MP and HP was statistically lower than that in the LP group (all *p*-values < 0.05) (Fig. 3D).

### Histological observation revealed better bone reconstruction in the HP group than in the MP and LP groups

**Fig. 4:**
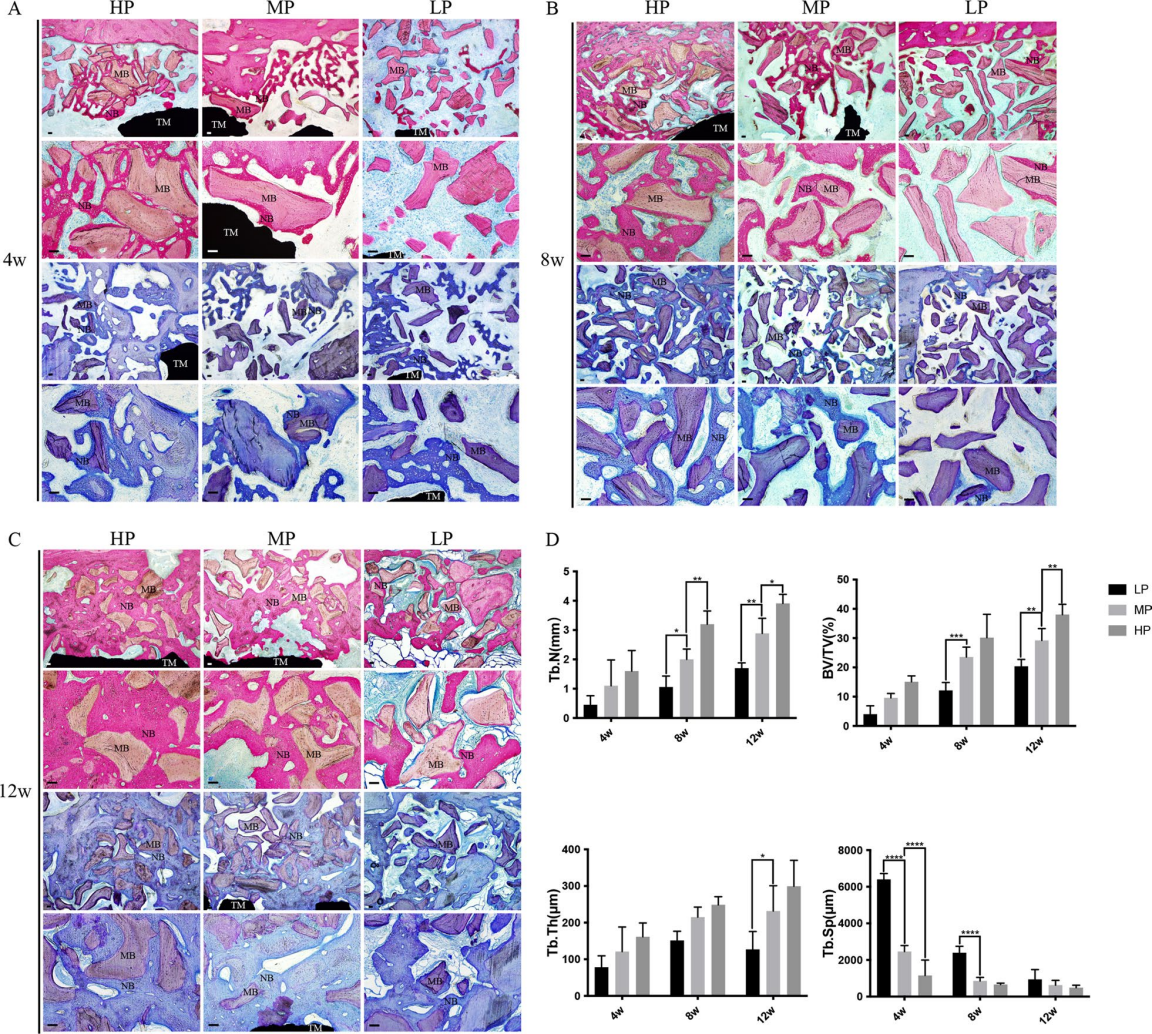
Histological observation of the repair effect of the titanium mesh with different porosities on bone defect. Basic fuchsin staining shows the new bone in dark pink and the mother bone in light pink, while toluidine blue staining shows the new bone in dark blue and the mother bone in light blue. **A** After 4 weeks, new bone formation was observed around the mother bone in the HP and MP groups, but to a lesser extent in the LP group. **B** After 8 weeks, a large area of new bone was formed in the HP group, new bone was also observed around the mother bone in the MP group, and to a lower extent in the LP group. **C** After 12 weeks, a large area of new bone was formed around the mother bone in the HP and MP groups, especially in the HP group where the bone defect area was filled. New bone formation was also observed in the LP group but with a large amount of fibrous tissues. **D** The Tb. N in the HP group was higher than that in the other two groups at 8 and 12 weeks. The BV/TV was higher than the other two groups at 12 weeks; Tb. TH in the MP group was higher than the LP group at 12 weeks, and Tb. SP was lower in the HP group at 4 and 8 weeks. The difference was statistically significant. MB, mother bone; NB, new bone; TM, titanium mesh. Scalebar = 50 μm

Histological observation revealed that the titanium mesh with HP showed good repair of the bone defects in vivo. The basic fuchsin staining shows the new bone in dark pink and the mother bone in light pink, while the toluidine blue staining shows the new bone in dark blue and the mother bone in light blue. After 4 weeks, a new bone was formed around the mother bone in the HP and MP groups and to a lower extent in the LP group (Fig. 4A). After 8 weeks, the HP group had a large area of new bone and the MP group showed a new bone area around the mother bone, while the LP group showed a lower extent of new bone formation (Fig. 4B). After 12 weeks, a large area of new bone was formed around the mother bone and the bone defect area was filled in the HP and MP groups, though it was more evident in the HP group. In the LP group, a new bone area was formed but with a large amount of fibrous tissue (Fig. 4C). The Tb. N in the HP group was higher than that in the other two groups at 8 and 12 weeks, the BV/TV was higher in the HP group than that in the other two groups at 12 weeks, the Tb. Th was higher in the HP group than that in the LP group at 12 weeks, and the Tb. Compared to the other groups, the SP was lower in the HP group at 4 and 8 weeks. These differences were all statistically significant (Fig. 4D).

## Discussion

In oral implant surgery, the titanium mesh plays an important role in the bone reconstruction of patients with large area or multiple tooth position jaw defects. The titanium mesh acts as a mechanical barrier membrane to provide sufficient low pressure space for the formation of new bone in alveolar ridge incremental surgery [14, 15]. It essentially acts as a “tent” that allows the regeneration and repair of large-area bone defects. Recent studies have shown that the clinical effect of its combined application with absorbable biofilm is better as the biofilm prevents the rapid growth of soft tissue [16]. Indeed, the bone increment effect of this treatment is better than the clinical effect of using biofilm alone [17–19]. In this study, we designed a 3D printed personalized titanium mesh. With the development of biomechanics and CBCT 3D imaging technology, we virtually reconstructed a 3D model of the bone defect through forward and reverse software and designed a personalized titanium mesh model. The 3D printed titanium mesh has many advantages. First, it can reconstruct and design the alveolar ridge shape of the defect. It also ensures the reconstruction of aesthetics and function after implantation. Second, titanium meshes that are suitable for bone and jaw shape defects can be printed out before the operation, which eliminates the need for the operator to adjust the shape of the titanium mesh and shortens the operation time. Third, the personalized titanium mesh can reduce the probability of postoperative titanium mesh exposure. The osteogenic effect of unexposed titanium mesh is better than that of exposed mesh [13]. This could be mainly attributed to the lack of keratinized mucosa in the oral cavity of animals, where the frenum and muscles in the mouth are often attached to the alveolar bone [13]. Thus, the personalized titanium mesh acts as a substitute for the oral mucosa. Compared with the bone defect in the simple horizontal direction, horizontal and vertical bone defects are more likely to lead to postoperative exposure of the titanium mesh, resulting in the amount of bone formation being significantly reduced [20–23].

Several criteria were considered at the design stage of the titanium mesh, such as good mechanical properties; high compressive strength that can provide stable spatial support during the osteogenesis process; appropriate elastic modulus and plasticity that can reduce the pressure on the mucosa; and good corrosion resistance. These characteristics are key to achieve stable osteogenesis [24, 25]. The factors that were considered mainly included the material, thickness, pore size, shape, and porosity of the titanium mesh. These factors are critical to the strength of the titanium mesh and to provide sufficient blood supply to the bone defect reconstruction site [26].

Previous studies have shown that pure titanium is an ideal material for creating the titanium mesh because of its low density, high strength, corrosion resistance, good biocompatibility, and close elastic modulus to the cortical bone [27]. Clinically, a titanium mesh with appropriate thickness should be selected according to the range and location of jaw defects and its mechanical properties should meet the clinical needs. Theoretically, the thicker the titanium mesh, the better the mechanical properties. However, while considering the mechanical properties of the titanium mesh, the stimulation of the thickness of the titanium mesh to the mucosa should also be considered to reduce the possibility of exposure. The thickness of the titanium mesh used in this study was in accordance with the thickness of the titanium mesh used in previously published studies [13]. A titanium mesh with a thickness of 0.4 mm can not only ensure sufficient strength but also reduce its thickness [28]. In this experiment, the titanium mesh was designed as a single cell structure composed of round and spindle holes. The diameter of the round holes was fixed but the size of the spindle holes was controlled by the porosity. In a previous study, human osteoblasts were cultured on the surface of three kinds of porous titanium alloy scaffolds with different pore shapes (cubic, conical, and diagonal). The results showed that the metabolic activity of osteoblasts on the conical microporous scaffolds was significantly higher than that on the other two scaffolds. However, no significant difference was found between the metabolic activity of osteoblasts on the surfaces of the cubic and diagonal structures [29]. Thus, different pore shapes can affect the osteogenic ability of porous implants, but there is no clear consensus on the optimal pore shape and its mechanism for regulating the bone induction performance of implants, which may be related to the difficulty in controlling the pore shape in experiments. The pore size has always been the focus of titanium mesh designs. The ideal pore size should not only facilitate the transportation and exchange of substances between cells and provide a convenient environment for the proliferation and migration of osteoblasts and the growth of nerve vessels but also ensure sufficient strength to withstand bone stress. In general, a very small pore size will restrict the growth of cells, hinder the transportation of blood and nutrients, and lead to poor growth of osteoblasts. On the other hand, a very large pore diameter reduces the compressive property and strength of the implant material. Therefore, to determine the most suitable pore size for osteogenesis, it is necessary to find the best balance between the mechanical properties and biocompatibility of materials [30–32].

The shape of pores in the 3D printed titanium mesh is often irregular; thus, porosity is used to evaluate the volume proportion of pores [33]. Porosity is the percentage of the pore volume of a material and the total volume of the material in the natural state. It plays the same role as the pore size. The porosity of an implant can affect free movement ability and exchange ability of substances between different pores and affect the bone growth level of the material [34, 35]. In this study, the titanium mesh with HP shows better osteogenic effects in imaging and histology than the mesh with LP under the same conditions of material type, pore size, and thickness. Micro-CT is an important imaging method to evaluate the osseointegration effect of implants. Micro-CT can display the bone mineral density and the microstructure of 3D bone trabeculae in the bone defect area. Compared with the traditional histological analysis method, it causes less trauma and can directly calculate the late trabecular bone structural parameters through micro-CT analysis software, mainly through the Tb. N, Tb. SP, and Tb. Th parameters. The Tb. N, the maturity of bone tissue, and the bone density are positively correlated with BS/BV and BV/TV [36, 37]. Methylene blue staining and toluidine blue staining are commonly used to observe the formation of new bone. In this study, we can clearly identify the boundary between the mother bone and the new bone in the bone defect area of each group. After 4 weeks, a small area of new bone appeared in the HP group, while a large number of fibrous tissue still surrounded the mother bone in the LP group. After 8 weeks, the new bone in the HP group connected the bone debris. In the 12-week HP group, the bone defect area was basically filled with new bone and mother bone. In addition, the bone parameter analysis data were consistent with the results of the micro-CT. In this study, the HP group showed a faster rate of new bone formation and excellent bone regeneration effect. In principle, the closer the porosity of porous implants is to the porosity of human cancellous bone (70–90%), the more favorable it is for bone growth. If the porosity is too high, the compressive property and strength of the implant will be reduced, and it would be difficult to bear the stress of the bone, resulting in a shortened service life. If the porosity is too low, it will hinder the material exchange of cells and affect the osteogenic ability [38, 39]. The porosity of the titanium mesh is not certain, but is closely related to the design of the pore size and shape of the titanium mesh. In this study, we designed the porosity of the titanium mesh to be 55%, 62%, and 68% in the LP, MP, and HP groups, respectively, set by researchers after 3D finite element analysis in the early stage to ensure that the titanium mesh has sufficient strength. The 3D printed titanium mesh with HP in a certain range may have more advantages than a titanium mesh with LP in repairing large-area bone defects. In addition, Tamaddon et al. developed a porous titanium scaffold with a porosity of 72% by using 3D printing technology and inoculated sheep bone marrow mesenchymal stem cells (BMSCs) on its surface for in vitro culture. The cells had good adhesion ability on the surface of the scaffold [40]. In another study, three kinds of porous titanium alloy scaffolds with porosities of 15.0% ± 2.9, 37.9% ± 4.0, and 70.0% ± 3.5 were prepared and MG-63 cells were planted on their surfaces through 3D printing technology. The cells on the surface of the three scaffolds had a high survival rate, but the cells in the 70.0% ± 3.5 HP group had the highest survival rate [41]. Xu et al. prepared porous titanium alloy scaffolds with porosities of 40% and 70%, inoculated rabbit BMSCs for culture, and used dense titanium alloy as a control. The cells in the 40% and 70% porosity groups proliferated faster on the surface of the material, and the cells in the 70% porosity group grew from the edge to the pores in a shorter time with more tight connections [42].

Although the traditional titanium mesh can make up for the shortcomings of collagen barrier membrane, it needs to be bent manually during the operation to prolong the operation time, and its sharp edges are easy to cause mucosal damage, which also increases the risk of titanium mesh exposure. Compared with collagen membrane and traditional titanium mesh, 3D printing personalized titanium mesh has good mechanical properties, which can not only provide stable and sufficient osteogenic space for bone regeneration in horizontal and vertical directions but can also perfectly fit the original contour of the alveolar bone, prevent the compression of the mucosa from affecting the stability of the bone graft material, reduce the number of retaining nails, and improve patient satisfaction. In addition, the amount of bone incremental materials can be accurately estimated according to the needs of implant surgery to avoid wastage of bone incremental materials or insufficient bone graft [43]. Therefore, 3D printing personalized titanium meshes are considered to be more accurate and minimally invasive. However, a personalized titanium mesh has its limitations, such as titanium mesh exposure. There is no systematic evidence on the influencing factors and improvement measures of titanium mesh exposure. Compared with the traditional bone increment method, the personalized titanium mesh can achieve accurate bone increment; however, the final amount of bone formation is still different from the expected design. Thus, methods to improve the accuracy of bone increment need to be further explored.

## Conclusion

This study shows that the titanium mesh is an important auxiliary tool to repair large-area bone defects in oral implant surgery. Under similar conditions of material, pore size, shape, and thickness, a 3D printed titanium mesh with a higher porosity in a certain range may have more advantages than a titanium mesh with a lower porosity for repairing large-area bone defects. The findings of our study thus provide a reference for designing the porosity of titanium meshes used in oral implant surgery.

## Data Availability

The data sets used and/or analyzed during the current study are available from the corresponding author upon reasonable request.

## Abbreviations

BS/BV: Bone surface/bone volume ratio
BV/TV: Bone volume/total volume ratio
CAD: Computer-aided design
CAM: Computer-aided manufacturing
CT: Computed tomography
HP: High porosity
LP: Low porosity
MP: Medium porosity
Tb. N: Trabecular number
Tb. SP: Trabecular separation
Tb. Th: Trabecular thickness
